# Smoking status and attractiveness among exemplar and prototypical identical twins discordant for smoking

**DOI:** 10.1098/rsos.161076

**Published:** 2017-12-13

**Authors:** Andrew L. Skinner, Andy Woods, Christopher J. Stone, Ian Penton-Voak, Marcus R. Munafò

**Affiliations:** 1 MRC Integrative Epidemiology Unit (IEU) at the University of Bristol, Bristol, UK; 2 School of Experimental Psychology, University of Bristol, Bristol, UK; 3 United Kingdom Centre for Tobacco Control Studies, University of Bristol, Bristol, UK; 4 Crossmodal Research Laboratory, University of Oxford, Oxford, UK

**Keywords:** smoking, status, facial, attractiveness, identical twins

## Abstract

Smoking is associated with negative health of skin and increased signs of facial ageing. We aimed to address two questions about smoking and appearance: (1) does facial appearance alone provide an indication of smoking status, and (2) how does smoking affect the attractiveness of faces? We used faces of identical twins discordant for smoking, and prototypes made by averaging the faces of the twins. In Task 1, we presented exemplar twin sets and same sex prototypes side-by-side and participants (*n* = 590) indicated which face was the smoker. Participants were blind to smoking status. In Task 2 a separate sample (*n* = 580) indicated which face was more attractive. For the exemplar twin sets, there was inconclusive evidence participants selected the smoking twin as the smoker more often, or selected the non-smoking twin as the more attractive more often. For the prototypes, however, participants clearly selected the smoking prototypes as the smoker more often, and the non-smoking prototypes as the more attractive. Prototypical faces of smokers are judged more attractive and correctly identified as smokers more often than prototypical faces of matched non-smokers. We discuss the possible use of these findings in smoking behaviour change interventions.

## 1. Introduction

Cigarette smoking is associated with negative health and increased ageing of skin [1–4]. Several studies have indicated that smoking accelerates the visible signs of ageing in faces [5–9]. The extent to which faces age, however, is also governed by intrinsic factors such as age, sex and genetic disposition to ageing [10,11]. In order to explore the effects of smoking on facial appearance, it is therefore necessary to control for variations in these intrinsic individual differences.

Identical twins provide us with exactly this level of control. Twin studies have been used extensively to explore the heritability of smoking behaviour, indicating that somewhere between 45% and 85% of the variability in smoking initiation and persistence can be explained by genetic differences between individuals [12–14]. Because identical twins share nearly all their genetic material, and some aspects of their environment (e.g. parenting, cultural background, education), differences between them can be attributed to non-shared environmental effects, including differences in lifestyle behaviours such as smoking.

Between 2007 and 2010, researchers at Case Western Reserve University captured photographs of faces and detailed medical and lifestyle histories of sets of identical twins from the United States [15]. Of these, a number of sets were discordant for smoking: one twin was a smoker and the other was not (or had smoked at least 5 years less). This provides images of faces of individuals that differ in smoking status, but that are matched in terms of age, sex, and genetically determined ageing.

Okada *et al.* [15] explored differences in facial appearance between these smoking and non-smoking twins, and observed greater signs of age-related facial features (e.g. lower lid bags, malar bags, nasolabial folds, lip wrinkles and jowls) in the smoking twins. In a similar study of identical twins from Japan, Ichibori *et al.* [7] also found increased signs of facial ageing with smoking, with higher scores for facial texture and wrinkles in smokers.

In this study, we used these identical twins that are discordant for smoking to explore two further aspects of smoking and facial appearance: (1) does facial appearance alone provide an indication of smoking status and (2) how does smoking affect the attractiveness of faces?

One issue when comparing facial appearance within individual twin sets is that slight variations in lighting, pose, and expression can affect facial appearance. Subtle variations in smile, for example, can result in changes to wrinkles that can influence judgements about ageing [15]. To address this issue, we produced prototype smoking and non-smoking faces, and included comparisons of these prototypes in our tasks. A prototype is made by averaging the shape, texture and colour of the faces of the group of interest. The averaging process removes idiosyncratic variations in facial appearance and variations in lighting, pose and expression, and distils the common visual characteristics of the exemplar faces from which it was produced. The use of prototypes was planned from the beginning of this study (which is hopefully clear from their inclusion in the tasks reported, alongside the exemplars), but unfortunately was omitted from the pre-registered protocol for this study by error. We have therefore been conservative and not attributed the findings relating to prototypes reported here to pre-registered analyses.

## 2. Methods

### 2.1. Participants

Participants were recruited using the Prolific Academic crowdsourcing platform (https://www.prolific.ac/). To prevent issues with demand characteristics, separate groups of participants were recruited for each task. No age or location restrictions were imposed on recruitment profiles.

### 2.2. Design

Both tasks were 2 alternative forced choice (2AFC) designs, with actual smoking status (smoker, non-smoker) as the independent variable, and ‘perceived smoking status’ (Task 1) and ‘more attractive’ (Task 2) as dependent variables.

In each task, there were 25 trials. In each trial, one of the 25 sets of faces (23 twin sets and male and female prototypes) was displayed side-by-side. The left–right order of smoker and non-smoker was randomized on a trial-by-trial basis. The order of sets presentation was randomized for each participant.

The protocol for this study was pre-registered with the Open Science Framework (https://osf.io/bp3ag/). The use of exemplar twin sets and analyses of these was included in this protocol, but the use and analyses of prototypes was unfortunately omitted by error.

### 2.3. Materials

High quality photographic images of faces of identical twins were supplied by the Department of Plastic Surgery at Case Western University. They were originally captured by the Case Western team from identical twins attending the Twin Days festival in Ohio in the United States between 2007 and 2010. Twins also completed a questionnaire about their medical and lifestyle histories. From these, a number of sets of twins were identified that were discordant for smoking. These are sets in which one twin smoked and the other has never smoked, or had smoked at least 5 years less. Images of faces of 23 sets of identical twins discordant for smoking were made available for the current study (20 female, mean age 57 years; s.d., 10.7 years).

Availability of data describing other lifestyle factors likely to affect facial appearance [6] for the twins varied between twin sets, and are summarized in table 1.

**Table 1.** Additional lifestyle factors for twin sets.

factor (measure)	number of twinsets data available	smoking twins group value	non-smoking twins group value	statistical test of group difference	Bayes factor (paired sample *t*-test)
alcohol consumption (drinks/week)	15	M = 2.8	M = 2.0	*p *= 0.21^a^	0.54
BMI (kg m^−2^)	16	M = 26.7	M = 26.2	*p *= 0.59^a^	0.29
lifetime sun exposure (years)	18	M = 38.6	M = 39.1	*p *= 0.86^a^	0.25
use of sun protection (yes/no)	21	62%	62%	*p *= 0.99^b^	0.23
use of moisturizer (yes/no)	23	70%	78%	*p *= 0.50^b^	0.34

^a^Paired sample *t*-test. ^b^Chi-squared test.

### 2.4. Creating prototypes

**Figure 1.**
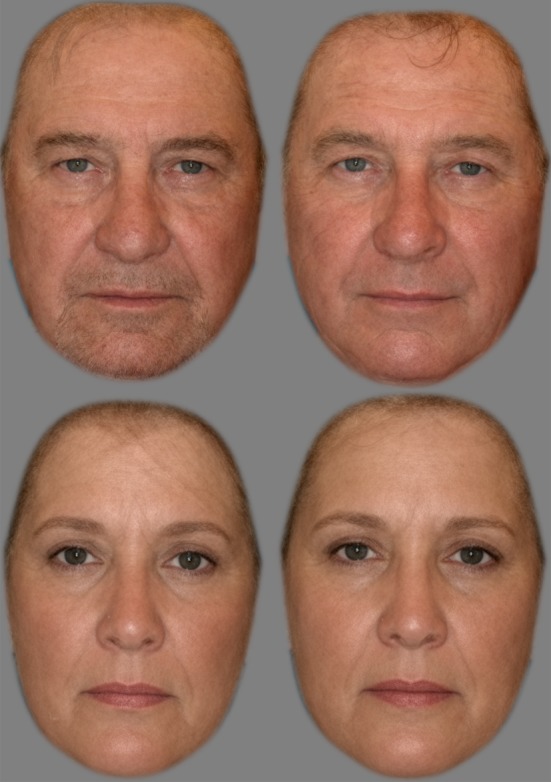
Prototype male (top) and female (bottom) smoking (left) and non-smoking (right) faces.

To create prototypes we followed an established procedure for making high quality prototype face stimuli (e.g. [16]), using the facial image manipulation software package PsychoMorph v.6 [17]. We produced four prototypes in total: male smoker, male non-smoker, female smoker, female non-smoker. These are shown in figure 1. To produce the prototype male smoking face, the face of each exemplar male smoking twin was loaded into PsychoMorph, and its features (e.g. corners of eyes and mouth) were identified using a number of fiducial markers. PsychoMorph's averaging function was then used to produce the male smoking prototype by averaging the shape, colour, and texture (using wavelet processing) of all the male smoking exemplar faces. Finally, the image of the prototype face was masked to remove detail of hair and clothing, using a Gaussian blur to mid-grey background. The process was repeated to produce a male non-smoking prototype, and female smoking and non-smoking prototypes.

The marking-up process was identical for all exemplar faces for all prototypes, and the averaging process was entirely automatic and involved no information about the individuals (e.g. their smoking status). This means it was extremely unlikely the appearance of the prototypes could have been systematically influenced by researchers, even with knowledge of the smoking status of the exemplar faces. The number of exemplar twin sets available for use for this study was limited, and three male twin sets were used to produce the male prototypes, and 20 female twin sets were used to produce the female prototypes.

### 2.5. Procedure

The tasks were authored in the Haxe (https://haxe.org/) based software Xperiment (http://www.xperiment.mobi), transpiled into JavaScript, and hosted on Xperiment's Internet application platform. Participants were recruited using crowdsourcing platform Prolific Academic, then directed to the Xperiment site. Each participant completed only one of the two tasks. They were free to complete the task on any device type (e.g. desktop, laptop, tablet).

After giving consent participants answered a brief set of demographic questions before beginning the task. In each trial they were presented with a set of prototypes (or twins) side-by-side and asked to judge either ‘Which person smokes cigarettes?’ (Task 1) or ‘Which person is more attractive?’ (Task 2). They responded by clicking or touching one of the faces. The faces remained on screen until the participant responded. Each task took approximately 5 min to complete, after which they were returned to Prolific Academic and reimbursed £0.50.

### 2.6. Statistical analysis

Responses in both tasks were scored as 0 for a response indicating the non-smoking face, and 1 for the smoking face. To visualize data, mean responses were calculated for each twin and prototype set by summing the participant responses and dividing by the total number of participants. A mean response value greater than 0.5 would therefore indicate a preference for a smoking face, and less than 0.5 a preference for a non-smoking face. Binomial confidence intervals (95%) were also calculated for each mean, using

$$\text{CI}=1.96\sqrt{\left(\frac{p(1-p)}{n}\right)},$$

where *p* = mean response and *n* = number of participant responses.

Data from tasks in which participants make judgements about visual stimuli are typically analysed *by participant*. In this approach, each participant's judgements are averaged across all stimuli in a category, then differences between participant means in categories are compared. This kind of analysis treats participants as random factors, but ignores the random effects due to stimuli. This limits the extent to which it is possible to generalize findings about stimuli [18,19]. However, it is also possible to analyse *by stimulus*. This treats stimuli as random factors, and ignores random effects from participants. In this case, mean judgements are computed for each stimulus, then means for stimuli are compared across categories. For facial attractiveness, consensus in judgements of attractiveness is generally high (hence a mean of several ratings represents a credible ‘ground truth’) [20], so this type of analysis is frequently performed.

We analysed responses to exemplars in both ways. Firstly, they were analysed by participant. A score for each participant was calculated by summing their responses to each of the exemplar twin sets, then dividing by the total number of twin sets. The mean and 95% confidence intervals of all participant scores were then calculated. A one-sample *t*-test compared all participant scores with a reference value of 0.5. To enable comparison with analyses by stimulus, we also performed a Bayesian one-sample *t*-test.

Secondly, exemplars were analysed by stimulus (twin set). The mean of all participant responses to each twin set was calculated, then the mean and 95% CIs were calculated for all twin sets. A one-sample *t*-test was performed, with reference value set to 0.5. This was performed for male and female participants separately. An independent sample *t*-test was used to test for differences between male and female participant responses. As the number of twin sets was small and this analysis likely to be underpowered, we also performed a Bayesian one-sample *t*-test, with reference set to 0.5, and Cauchy prior width set to 0.707.

To analyse responses to prototypes we performed exact binomial tests of mean responses, with reference values set to 0.5.

In determining an appropriate sample size, few comparable studies were available to guide selection of an appropriate effect size. One recent study [21] reported higher attractiveness ratings for faces of individuals with diets high in fruit and vegetables, with an effect size of 0.29, but this compared attractiveness ratings from 7-point scales, not proportions in a 2AFC task. We therefore took a cautious view and powered each task to detect effect sizes of 0.2 or greater for each sex of participant. We calculated that a total of 315 male and 315 female participants would be required to achieve 95% power at an alpha level of 5% in each task.

## 3. Results

As this study began before our laboratory had put in place procedures to support full open access to data, we unfortunately do not have consent to make the data for this study fully open. We are, however, able to make the data freely available on request from our institutional data repository (http://data.bris.ac.uk/data/), doi:10.5523/bris.tyg5f7au6rkb2nizyjyr7wqgj.

### 3.1. Participant details

Participants were excluded from the study if they failed to enter demographic information correctly (Task 1: 13 participants, 2%; Task 2: 12 participants, 2%), or if they did not complete the study (Task 1: 39 participants, 6%; Task 2: 23 participants, 4%).

After exclusions, 590 participants completed Task 1 (51% female, mean age 32.7 years, s.d. 11.3 years), and 580 participants completed Task 2 (50% female, mean age 31.6 years, s.d. 11.0 years).

Ideally, recruitment for both tasks would have compensated for exclusions and continued to the numbers identified in the sample size calculations. Unfortunately, it was not possible to configure the automated crowdsourcing and application platform used in this study to compensate for the various (sometimes unexpected) exclusions encountered. However, given the conservative nature of our sample size calculation, and the low number of exclusions, this would not have been an issue for the tasks reported here.

### 3.2. Exemplar analyses by participant

Task 1: Smoking twins were judged to be smokers more often than non-smoking twins by both male participants (mean response = 0.53, 95% CIs = 0.52, 0.54, *t*(284) = −5.52, *p* < 0.001) and female participants (mean response = 0.55, 95% CIs = 0.54, 0.56, *t*(294) = −8.29, *p* < 0.001). Overall, females were marginally more likely to judge smoking twins as smokers than males (*t*(578) = −2.14, *p* = 0.03). Bayesian analyses found extreme evidence to support the hypothesis that participants could correctly identify the smoking twins (BF_10_ males = 2.23e+4, BF_10_ females = 2.61e+12).

Task 2: There was evidence of a preference for non-smoking twins for both male participants (mean response = 0.44, 95% CIs = 0.43, 0.45, *t*(294) = 11.3, *p* < 0.001) and female participants (mean response = 0.44, 95% CIs = 0.43, 0.45, *t*(294) = 12.0, *p* < 0.001). There was no evidence of a difference between male and female participant responses (*t*(588) = 0.99, *p* = 0.32). Bayesian analyses found extreme evidence to support the hypothesis that participants find the smoking twins more attractive (BF_10_ males = 1.05e+22, BF_10_ females = 1.86e+24).

### 3.3. Exemplar analyses by stimulus

Task 1: There was no evidence either smoking or non-smoking twins were judged to be smokers by male participants (mean response = 0.53, CIs = 0.44, 0.62, *t*(22) = −0.68, *p* = 0.51) or female participants (mean response = 0.55, CIs = 0.45, 0.64, *t*(22) = −0.99*, p* = 0.33). Bayesian analyses found anecdotal evidence (the lowest acknowledged level of evidence [22]) that participants were unable to correctly identify the smoking twin (BF_10_ males = 0.4, BF_10_ females = 0.56).

Task 2: There was no evidence of a preference for either smoking or non-smoking twins in male participants (mean response = 0.44, 95% CIs = 0.35, 0.54, *t*(22) = 1.19, *p* = 0.24) or female participants (mean response = 0.43, 95% CIs = 0.34, 0.53, *t*(22) = 1.31, *p* = 0.20). Bayesian analyses found anecdotal evidence that participants had no preference for either twin (BF_10_ males = 0.71, BF_10_ females = 0.83).

**Figure 2.**
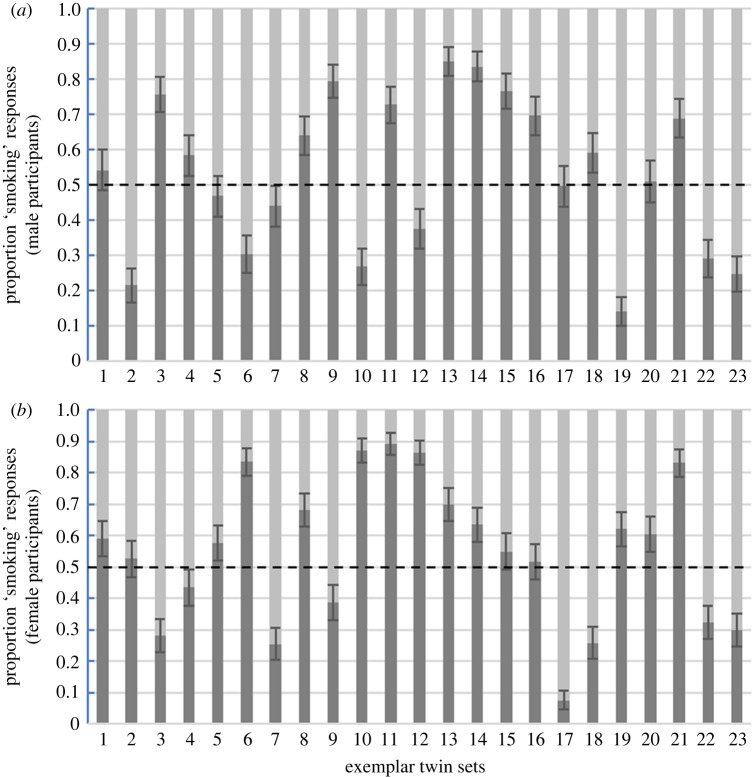
Task 1 exemplar results. Mean judgements of smoking status in male (1–3) and female (4–23) exemplars from male participants (*a*) and female participants (*b*). Responses were coded as non-smoker = 0, smoker = 1. Dotted line shows 0.5 level. Error bars are 95% binomial confidence intervals.

**Figure 3.**
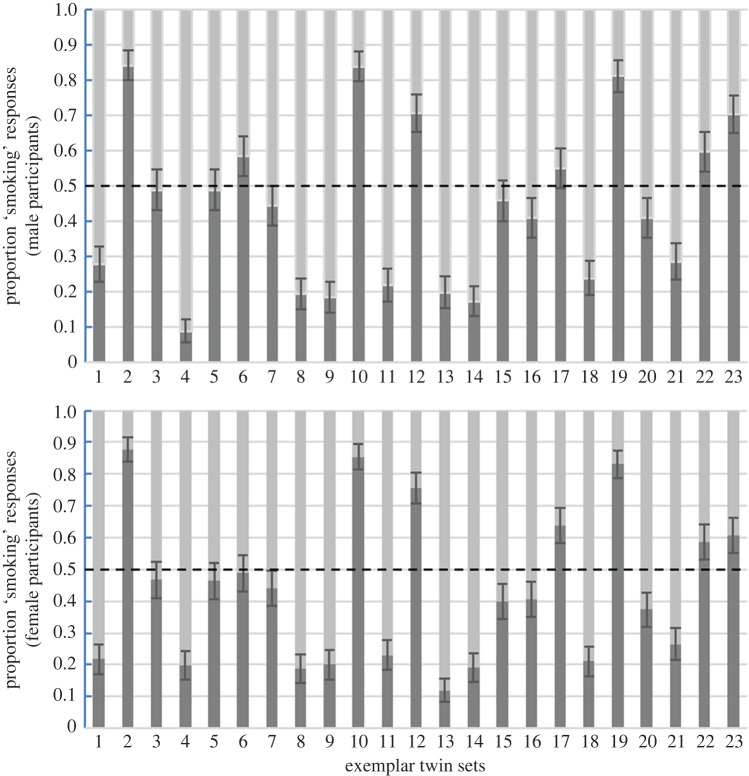
Task 2 exemplar results. Mean judgements of attractiveness in male (1–3) and female (4–23) exemplars from male participants (*a*) and female participants (*b*). Responses were coded as non-smoker = 0, smoker = 1. Dotted line shows 0.5 level. Error bars are 95% binomial confidence intervals.

Results for exemplars in Task 1 are illustrated in figure 2, and Task 2 in figure 3.

### 3.4. Prototype analyses

Task 1: An exact binomial test indicated male participants selected the smoking male prototype (mean response = 0.70, corresponding to 70%) and smoking female prototype (0.70, 70%) to be the smoker, and female participants selected the smoking male prototype (0.68, 68%) and the smoking female prototype (0.73, 73%) to be the smoker; all *p*s < 0.001.

Task 2: Exact binomial tests indicated male participants judged the male non-smoking prototype (mean response = 0.28, corresponding to 72%) and the female non-smoking prototype (0.34, 66%) as more attractive, and female participants judged the male prototype (0.32, 68%) and the female prototype (0.30, 70%) to be more attractive; all *p*s < 0.001.

**Figure 4.**
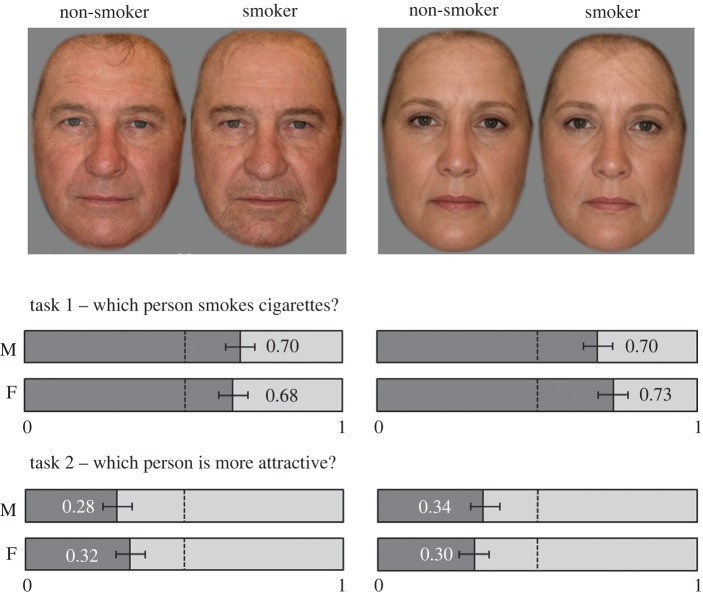
Mean judgements of smoking status and attractiveness in male and female prototypes from male (M) and female (F) participants. Responses were coded as non-smoker = 0, smoker = 1. Dotted vertical shows 0.5 level. Error bars are 95% binomial confidence intervals.

Results for prototypes in Tasks 1 and 2 are illustrated in figure 4.

## 4. Discussion

Findings for the exemplar twin sets were inconclusive. Analysing exemplar data by participant (pre-registered) found strong evidence that participants were able to correctly identify the smoker, and found the non-smoking twin more attractive. However, analyses by stimulus (pre-registered), arguably a more appropriate analysis when attempting to generalize findings about stimuli, provided very weak evidence that participants could not correctly identify which twin in the exemplar twin sets was the smoker, and that they had no preference for the appearance of either twin. From the plots of exemplar results, it is clear there was considerable variation in participant responses between exemplar sets. Previous research has highlighted that one difficulty when comparing facial appearance within individual twin sets is that small variations in lighting, pose, and expression can have significant effects of facial appearance [15]. These factors, which are difficult to measure accurately and control for, could potentially introduce noise in the types of tasks reported here.

To address this issue, we produced prototype smoking and non-smoking faces by averaging the faces of the smoking and non-smoking twins, and included comparisons of these in both tasks (not pre-registered). The averaging process used to produce the prototypes means these judgements were not based on individual physiognomy, or influenced by variations in factors such as lighting, pose and expression, but on the facial characteristics common to the smoking and non-smoking twins that were distilled by the averaging process. The use of sets of identical twins to produce these prototypes also means that differences in appearance between the smoking and non-smoking prototypes were not caused by intrinsic individual differences, including genetic disposition to increased ageing.

Participants were able to correctly identify which prototypes were formed from smokers, suggesting facial appearance provides some information about smoking status. Non-smoking prototype faces formed from the faces of non-smoking identical twins were judged more attractive than smoking prototype faces formed using the smoking twin siblings. This provides evidence that, while there will be some individual variation, the faces of people that smoke may age in a less attractive manner than those that do not.

Findings from both tasks from comparisons of prototypes, which address the influence of variations in lighting, pose, and expression on appearance, have potential for use in behaviour change interventions and public health messages aimed at smoking. The findings from Task 1 are of interest as knowledge that facial appearance provides information about smoking status may be of utility in behaviour change interventions or public health messages that use the social stigma of smoking to motivate quitting. Previous studies have demonstrated success with behaviour change interventions that use social stigmas as an incentive to quit (e.g. [23]). We acknowledge, however, there is some debate about the acceptability of these approaches [24].

One type of smoking behaviour change intervention targeted at young people is the use of applications illustrating the changes in facial appearance likely if they age as a smoker and a non-smoker. These work on the basis that young people are particularly sensitive to the potential negative effects smoking has on their attractiveness as they age [25]. Applications of this kind, which have been shown to be effective in changing behaviour and attitudes towards smoking in young adults [26–28], currently use transformations produced using the faces of unrelated groups of smokers and non-smokers. Basing these face transformations on prototypes produced from identical twins discordant for smoking instead would mean they illustrate changes in appearance that are truly representative of the effects of smoking, and address the potential criticism that they may reflect inadvertent differences in intrinsic individual differences such as genetic disposition to ageing between smoking and non-smoking groups.

One limitation of this study was the small number of exemplar twin sets available, and in particular, the small number of male exemplars available. This could potentially have been an issue in the construction of the male prototypes, which were made using just three sets of exemplar twins. This limits the extent to which the averaging process can remove idiosyncrasies, and increases the possibility that judgements are made on the basis of idiosyncratic elements from exemplar faces present in the prototype. However, we observed similar patterns of results for our male and female prototypes in both tasks, and our female prototypes were produced using a greater number of faces (20 sets), so this this does not appear to have been an issue here. When selecting the number of exemplars to use to produce prototypes for future studies of this kind, we are not aware of any firm guidelines for a minimum, but would suggest a good starting point is the work by Langlois and Roggman showing attractiveness ratings differ between prototypes made using 8 and 16 exemplars, but not between prototypes made using 16 and 32 exemplars [29]. The limited number of exemplar sets available here also meant we were unable to investigate questions such as how heaviness of smoking in the smoking twin affects judgements in these tasks, as these analyses would have been considerably underpowered. Future studies with more exemplars could consider exploring this and related questions.

A further limitation was that, although we aimed to recruit separate groups of participants for each task, and the requirement to complete only one task per participant was part of the instructions to participants, this was not enforced. This means it is possible some participants completed both tasks, which could potentially have introduced issues with demand characteristics. Future studies should ensure they use functionality in crowdsourcing platforms for making task availability contingent on completion or not of other tasks. Finally, there are other factors that could affect facial appearance, and these could have been confounding factors here. Data were available for some of the most important of these factors for some, but not all, twin sets. These data provided no evidence of differences between smoking and non-smoking twin groups in terms of alcohol consumption, BMI, lifetime sun exposure, use of sun protection and use of moisturizer.

## 5. Conclusion

Our results provide evidence that smoking may negatively impact facial appearance, and that facial appearance may provide information about smoking status. The findings, particularly those for the prototypes that represent the characteristic facial features of smokers and non-smokers, have the potential to be of utility in developing and improving smoking behaviour change interventions. Future studies and interventions should aim to use optimal numbers of exemplar twin sets, and gather comprehensive data describing additional factors that could affect appearance of twins and prototypes.

## Data accessibility

As this study began before our laboratory had put in place procedures to support full open access to data, we unfortunately do not have consent to make the data for this study fully open. We are, however, able to make the data freely available on request from our institutional data repository (http://data.bris.ac.uk/data/), doi:10.5523/bris.tyg5f7au6rkb2nizyjyr7wqgj.
