# Correction to ‘Smoking status and attractiveness among exemplar and prototypical identical twins discordant for smoking’

**DOI:** 10.1098/rsos.172231

**Published:** 2018-02-21

**Authors:** Andrew L. Skinner, Andy Woods, Christopher J. Stone, Ian Penton-Voak, Marcus R. Munafó

*R. Soc. open sci.* **4**, 161076. (Published 13 December 2017). (doi:10.1098/rsos.161076)

The penultimate sentence in the abstract is incorrect. It currently reads as follows:

‘Prototypical faces of smokers are judged more attractive and correctly identified as smokers more often than prototypical faces of matched non-smokers.’

The correct sentence is:

‘Prototypical faces of non-smokers are judged more attractive, and prototypical faces of smokers are correctly identified as smokers more often than prototypical faces of matched smokers/non-smokers.’

In Section 3.2, the last sentence should read: ‘Bayesian analyses found extreme evidence to support the hypothesis that participants found the non-smoking twins more attractive (BF_10_ males = 1.05e + 22, BF_10_ females = 1.86e + 24).’

In Section 3.4, the second paragraph should read: ‘Task 2: Exact binomial tests indicated male participants judged the male non-smoking prototype (mean response = 0.28, corresponding to 72%) and the female non-smoking prototype (0.34, 66%) as more attractive, and female participants judged the male non-smoking prototype (0.32, 68%) and the female non-smoking prototype (0.30, 70%) to be more attractive, all *p*s < 0.001.’

